# Survival and treatment patterns of patients with relapsed or refractory multiple myeloma in France — a cohort study using the French National Healthcare database (SNDS**)**

**DOI:** 10.1007/s00277-021-04522-y

**Published:** 2021-04-21

**Authors:** Cyrille Touzeau, Nadia Quignot, Jie Meng, Heng Jiang, Artak Khachatryan, Moushmi Singh, Vanessa Taieb, Jean-Vannak Chauny, Gaëlle Désaméricq

**Affiliations:** 1 Service d’hématologie clinique, Hôtel Dieu, Nantes, France; 2 CRCINA, INSERM, CNRS, Université d’Angers, Université de Nantes, Nantes, France; 3 Site de Recherche Intégrée sur le Cancer (SIRIC) « ILIAD », Nantes, France; 4 Certara Evidence & Access, Paris, France; 5 Certara Evidence & Access, Lorrach, Germany; 6 Certara Evidence & Access, London, UK; 7 Amgen Ltd, London, UK; 8 Amgen SAS, Boulogne-Billancourt, France

**Keywords:** Autologous stem cell transplant, France, Relapsed or refractory multiple myeloma, SNDS, Treatment patterns, Survival

## Abstract

Over the past decade, several drugs have been approved for the treatment of relapsed or refractory multiple myeloma (RRMM). This retrospective study, using the French National Healthcare database (SNDS), describes the treatment patterns and outcomes of patients with RRMM treated in real-world clinical practice in France. Patients were adults, with a diagnosis of multiple myeloma, who initiated second-line (2L) treatment approved for use in France between 2014 and 2018; this included bortezomib, carfilzomib, daratumumab, ixazomib, lenalidomide, or pomalidomide. Data were analyzed overall, by first-line (1L) autologous stem cell transplant (ASCT) status and by lenalidomide treatment status at 2L. In total, 12987 patients with RRMM were included in the study (mean age 69.5 years); 27% received an ASCT at 1L, and 30% received a lenalidomide-sparing regimen at 2L. Overall, and among the ASCT and non-ASCT subgroups, most patients received a bortezomib-based regimen at 1L, whereas lenalidomide-based regimens were most common at 2L. Among patients who received a lenalidomide-sparing regimen at 2L, this was most often a proteasome inhibitor-based regimen. Mortality rate was 26.1/100 person-years, and median (95% confidence interval) survival from 2L initiation was 32.4 (31.2–33.6) months. Survival differed by various factors, shorter survival was reported in the non-ASCT group, those receiving a lenalidomide-sparing regimen at 2L, older patients (≥ 70 years), and those with multiple comorbidities. This analysis provides insight into the real-world use of approved novel MM treatments and highlights an ongoing unmet need to improve outcomes, particularly for selected patient groups.

**Supplementary Information:**

The online version contains supplementary material available at 10.1007/s00277-021-04522-y.

## Introduction

Multiple myeloma (MM), characterized by clonal plasma cell proliferation in the bone marrow, is the second most common hematologic malignancy [1]. In 2018, the MM age-standardized incidence per 100000 was estimated at 4.3 in Europe [2], and the number of new cases in France was estimated to be 5500 [3].

Although advances in therapy have led to longer remission periods, MM is still considered an incurable disease: patients with MM will eventually experience relapse and/or their disease will become refractory to treatment [4, 5]. During the past decade, multiple phase 3 studies in relapsed or refractory MM (RRMM) demonstrated the clinical benefit of novel agents, including pomalidomide (immunomodulatory agent); ixazomib (oral proteasome inhibitor [PI]); carfilzomib (PI); daratumumab, elotuzumab, and isatuximab (monoclonal antibodies); and panobinostat (histone-deacetylase inhibitor) [6–16]. Based on these studies, the European Medicines Agency approved these agents for the treatment of RRMM [17]. The European Society for Medical Oncology (ESMO) guidelines mainly recommend using these novel agents in doublet and triplet combination regimens that include a corticosteroid (dexamethasone or prednisone) [4, 18]. However, it remains unclear to what extent guideline recommendations translate into clinical practice [17], and there is relatively little current evidence on real-world treatment patterns for RRMM in France [19, 20], Europe [1, 21–24], or indeed elsewhere [17, 25].

Given the rapidly evolving treatment landscape, the aim of this study was to use the latest available data (2014 to 2018) from the “Système National des Données de Santé” (SNDS, for French National Healthcare database) to describe the RRMM treatment patterns in France, including recently approved novel therapies. Survival outcomes were also assessed as an exploratory analysis.

## Methods

### Data source

This was a retrospective observational cohort study that used data obtained from the SNDS database, which includes claims data for more than 65 million individuals (including 50 million adults), from birth (or immigration) to death, and is highly representative of the French population, covering 99% of the total population [26]. The SNDS database contains several datasets linked together via a unique patient ID (social security number). These datasets include the “Système National d’Information Inter-Régime de l’Assurance Maladie” (SNIIRAM, national information system for health insurance dataset), which contains demographic and administrative patient data (e.g., age, sex, and place of residence); healthcare visits and procedures reimbursed (e.g., medicines, medical procedures, medical devices, lab tests); and date of death. Data from hospitals and other healthcare facilities are extracted from the “Programme de Médicalisation des Systèmes d'Information” (PMSI, French National Hospital Informatics database), which includes inpatient data such as medical information, related diagnosis (based on International Classification of Diseases, 10th Revision, Clinical Modification [ICD-10-CM] codes), medical procedures, imaging, external visits, external procedures performed, expensive medicines, and implantable devices. Causes of death are also extracted for both inpatients and outpatients from the Epidemiological Center for the Medical Causes of Death database (CépiDc).

### Study design

Patients were eligible for inclusion if they were adults (≥ 18 years) with a diagnosis of MM and had received at least one dose of an approved novel MM treatment of interest (bortezomib, carfilzomib, daratumumab, ixazomib, lenalidomide or pomalidomide) between 2014 and 2018 (Supplementary material 1). Patients with evidence of second-line (2L) treatment (defined as at least one previous line of therapy, either unspecified or including any drug of interest) were considered to have RRMM (Supplementary material 1). Data were extracted for a 10-year period (01/01/2009–12/31/2018). The index date was defined as the date of initiation of the novel MM treatment of interest as a 2L treatment for RRMM. A baseline period was defined as the time period preceding the index date, up to study start or date of first diagnosis with MM (if MM was diagnosed after 01/01/2009). Patients were followed up from the index date until the last available information in the datasets, death or end of study (12/31/2018), whichever was first.

### Baseline characteristics

Demographics, clinical characteristics, and history of autologous stem cell transplant (ASCT) were assessed during the baseline period. Codes for identifying specific procedures and treatments are presented in Supplementary material 2. The Charlson comorbidity index (CCI) was retrospectively estimated according to an algorithm adapted from Bannay [27]. Frailty scores could not be estimated due to the unavailability of some key variables, such as laboratory-based biomarkers [28–30].

### Exposures and outcomes

Definitions of treatment regimens and lines of therapy were based on the algorithm described by Palmaro et al. [31], using the French national healthcare insurance database (SNIIRAM). The treatment regimens were also in line with ESMO guidelines [4]. It was assumed that supporting therapies, such as dexamethasone and prednisone, were given in combination with each treatment even though they are not visible within the SNDS dataset. Treatments were assigned to mutually exclusive regimens (Supplementary material 3). Mortality was estimated as the number of patients who died by the end of follow-up and rate was reported per person-years of follow-up.

### Statistical analysis

Results for treatment patterns and clinical characteristics were summarized by calculating the frequency and percentages for categorical variables and mean, standard deviation (SD), median, and interquartile ranges (IQR) for continuous variables. Overall survival was summarized using Kaplan-Meier methodology. The analyses were conducted overall, by ASCT status at first-line (1L) treatment and by lenalidomide status at 2L (defined as 2L regimens including lenalidomide, either doublets or triplets). Further analysis by lenalidomide status was conducted using the Cox regression model for all-cause mortality, adjusted by age at initiation of 2L treatment, time from MM diagnosis to initiation of 2L treatment, 1L treatment received, sex, ASCT status at 1L, and presence of comorbidities (hypertension, dementia, diabetes mellitus, moderate to severe renal disease or any tumor). All statistical analyses were conducted using SAS® Enterprise Guide version 7.15. Graphical representation was carried out using R version 3.6.2.

## Results

### Patient disposition

**Fig. 1:**
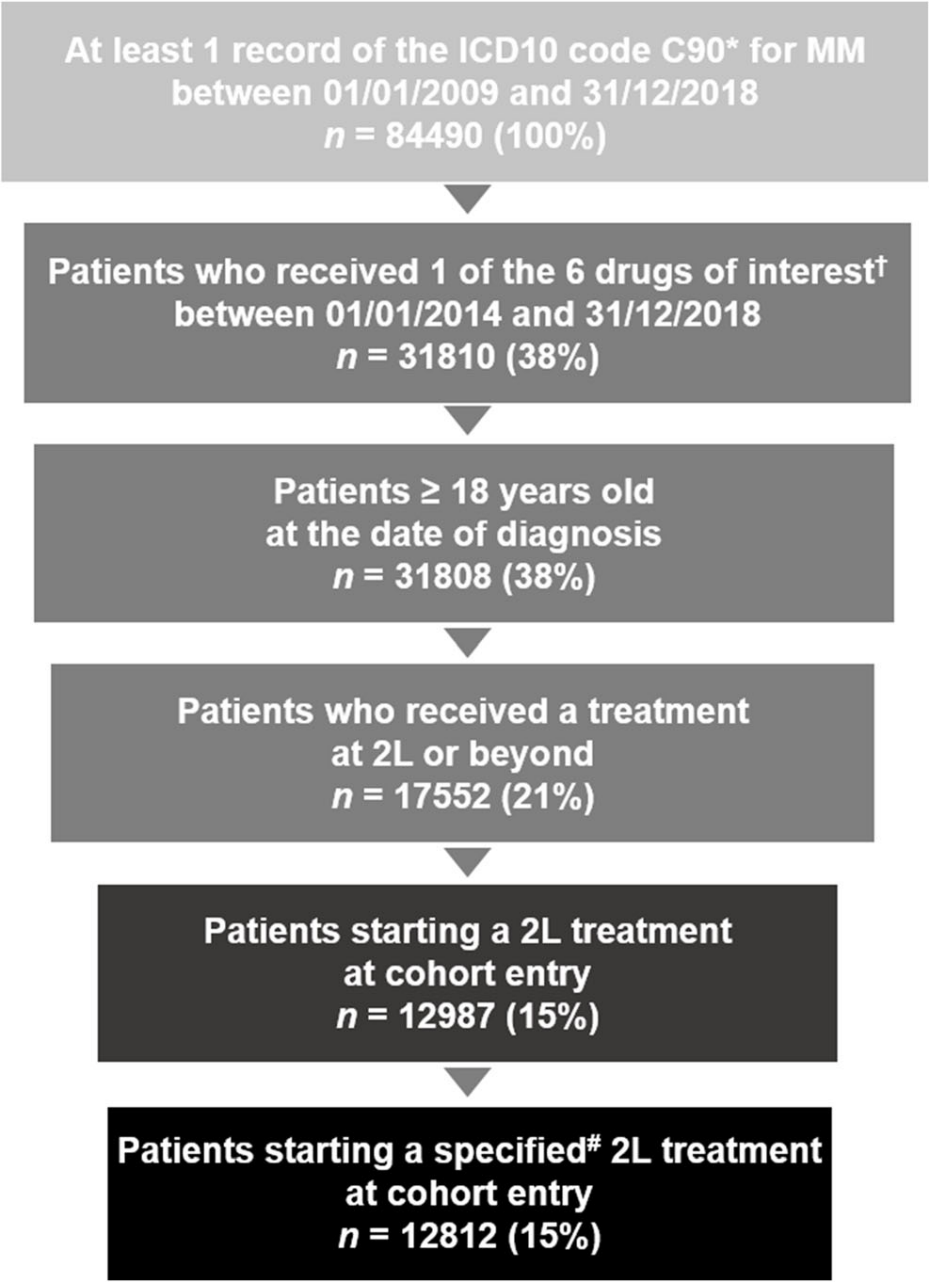
**Patient disposition.** ^*^Either principal, associated or related diagnosis code.^. †^Bortezomib, carfilzomib, daratumumab, ixazomib, lenalidomide or pomalidomide. ^**#**^175 patients received an unspecified chemotherapy treatment at 2L. *Abbreviations: ICD10*, International Classification of Diseases, 10th Revision; *MM*, multiple myeloma; *2L*, second line

Between 2014 and 2018, 12987 patients with RRMM were treated with at least one drug of interest, started a 2L treatment, and were included in the study cohort (Fig. 1).

### Baseline characteristics

The mean (SD) age at index date was 69.5 (10.6) years; 54% of patients (*n* = 7028) were male.

### Baseline characteristics by ASCT status at 1L

Twenty-seven percent of patients (*n* = 3454) received an ASCT at 1L (ASCT subgroup), and 73% of patients (*n* = 9533) did not (non-ASCT subgroup). Patients in the ASCT subgroup were younger than those in the non-ASCT subgroup (mean [SD] age 60.6 [7.6] years vs 72.7 [9.7] years) (Table 1). The proportion of patients who had at least one comorbidity was higher in the non-ASCT subgroup than in the ASCT subgroup (47% vs 32%). Comorbidities commonly reported for patients with RRMM were more frequent in the non-ASCT subgroup than in the ASCT subgroup (hypertension, 48% vs 31%; diabetes mellitus, 14% vs 10%; moderate to severe renal disease, 12% vs 5%). Similarly, patients in the non-ASCT subgroup had a higher CCI than those in the ASCT subgroup (mean [SD] CCI: 1.6 [2.5] vs 0.9 [2.0]).

**Table 1:** Baseline characteristics by 1L ASCT status

1L ASCT status	ASCT	Non-ASCT	All^a^
Number of patients (*n*, %)	3454 (27%)	9533 (73%)	12 987 (100%)
Age at index date^b^ (years)			
Mean (SD)	60.6 (7.6)	72.7 (9.7)	69.5 (10.6)
≤ 60 (*n*, %)	1462 (42%)	1052 (11%)	2514 (19%)
61–65 (*n*, %)	930 (27%)	791 (8%)	1721 (13%)
66–70 (*n*, %)	917 (27%)	1568 (16%)	2485 (19%)
71–75 (*n*, %)	139 (4%)	2062 (22%)	2201 (17%)
76–80 (*n*, %)	≤ 5 (0%)	2059 (22%)	2064 (16%)
> 80 (*n*, %)	≤ 5 (0%)	2001 (21%)	2002 (15%)
Sex (*n*, %)			
Male	2042 (59%)	4986 (52%)	7028 (54%)
Female	1412 (41%)	4547 (48%)	5959 (46%)
Time from MM diagnosis to index date (months)			
Mean (SD)	38.1 (32.9)	36.0 (41.9)	36.6 (39.7)
Charlson comorbidity index			
Mean (SD)	0.9 (2.0)	1.6 (2.5)	1.4 (2.4)
0 (*n*, %)	2355 (68%)	5050 (53%)	7405 (57%)
1 (*n*, %)	452 (13%)	1439 (15%)	1891 (15%)
2 (*n*, %)	270 (8%)	1114 (12%)	1384 (11%)
> 2 (*n*, %)	377 (11%)	1930 (20%)	2307 (18%)
Comorbidities (*n*, %)^c^			
Hypertension	1073 (31%)	4573 (48%)	5646 (43%)
Dementia	193 (6%)	802 (8%)	995 (8%)
Diabetes mellitus	352 (10%)	1354 (14%)	1706 (13%)
Moderate to severe renal disease	163 (5%)	1131 (12%)	1294 (10%)
Any tumor (including lymphoma and leukemia except for malignant neoplasm of skin)	186 (5%)	883 (9%)	1069 (8%)
Metastatic solid tumor	249 (7%)	939 (10%)	1188 (9%)
Follow-up time from index date (months)			
Mean (SD)	20.7 (15.4)	17.8 (15.2)	18.5 (15.3)
Median (IQR)	17.7 (7.7–31.0)	13.7 (5.1–27.1)	14.6 (5.7–28.3)

^a^Number of eligible patients who initiated a 2L treatment between 2014 and 2018 (1L ASCT status is defined as having ASCT at 1L or not)

^b^Index date was defined as the date at which the patient entered the cohort (= at 2L treatment initiation)

^c^Including all patients with at least one comorbidity; note that patient numbers across comorbidities do not sum to the total number of patients because one patient could have multiple comorbidities

*Abbreviations: ASCT,* autologous stem cell transplant; *IQR,* interquartile range; *MM,* multiple myeloma; *SD,* standard deviation; *1L,* first line; *2L,* second line

### Baseline characteristics by lenalidomide status at 2L

Seventy percent of patients (*n* = 8940) received a lenalidomide-based regimen at 2L (lenalidomide subgroup), and 30% of patients (*n* = 3872) did not (lenalidomide-sparing subgroup). Patients treated with a lenalidomide-based regimen at 2L were slightly younger than those treated with lenalidomide-sparing regimens (mean [SD] age 69.3 [10.3] vs 70.3 [11.2] years) and had a shorter time from first recorded MM diagnosis to 2L treatment initiation regimens (mean [SD] time 33.6 [35.1] vs 43.5 [47.8] months) (Table 2).

**Table 2:** Baseline characteristics by 2L lenalidomide status

2L Lenalidomide status	Lenalidomide-based regimen	Lenalidomide-sparing regimen	All^a^
Number of patients (*n*, %)	8940 (70%)	3872 (30%)	12 812 (100%)
Age at index date^b^ (years)			
Mean (SD)	69.3 (10.3)	70.3 (11.2)	69.6 (10.6)
≤ 60 (*n*, %)	1730 (19%)	720 (19%)	2450 (19%)
61–65 (*n*, %)	1197 (13%)	470 (12%)	1667 (13%)
66–70 (*n*, %)	1773 (20%)	681 (18%)	2454 (19%)
71–75 (*n*, %)	1591 (18%)	594 (15%)	2185 (17%)
76–80 (*n*, %)	1395 (16%)	661 (17%)	2056 (16%)
> 80 (*n*, %)	1254 (14%)	746 (19%)	2000 (16%)
Sex (*n*, %)			
Male	4885 (55%)	2034 (53%)	6919 (54%)
Female	4055 (45%)	1838 (47%)	5893 (46%)
Time from MM diagnosis to index date (months)			
Mean (SD)	33.6 (35.1)	43.5 (47.8)	36.6 (39.6)
Hematopoietic stem cell transplantation prior to index date (*n*, %)			
Yes	2912 (33%)	487 (13%)	3399 (27%)
No	6028 (67%)	3385 (87%)	9413 (73%)
Lenalidomide exposure prior to index date (*n*, %)			
Yes	253 (3%)	2027 (52%)	2280 (18%)
No	8687 (97%)	1845 (48%)	10 532 (82%)
Charlson comorbidity index			
Mean (SD)	1.4 (2.4)	1.5 (2.4)	1.4 (2.4)
0 (*n*, %)	5195 (58%)	2113 (55%)	7308 (57%)
1 (*n*, %)	1343 (15%)	525 (14%)	1868 (15%)
2 (*n*, %)	899 (10%)	462 (12%)	1361 (11%)
> 2 (*n*, %)	1503 (17%)	772 (20%)	2275 (18%)
Comorbidities (*n*, %)^c^			
Hypertension	3874 (43%)	1707 (44%)	5581 (44%)
Dementia	688 (8%)	291 (8%)	979 (8%)
Diabetes mellitus	1182 (13%)	508 (13%)	1690 (13%)
Moderate to severe renal disease	740 (8%)	536 (14%)	1276 (10%)
Any tumor (including lymphoma and leukemia except for malignant neoplasm of skin)	723 (8%)	325 (8%)	1048 (8%)
Metastatic solid tumor	827 (9%)	340 (9%)	1167 (9%)
Follow-up time from index date (months)			
Mean (SD)	20.3 (15.2)	14.4 (14.8)	18.5 (15.3)
Median (IQR)	17.1 (7.8–30.3)	9.1 (2.5–22.5)	14.6 (5.7–28.3)

^a^Number of eligible patients who initiated a 2L treatment identifiable in the database between 2014-2018 (175 patients received an unspecified treatment at 2L, 2L Lenalidomide status is known as regimen including lenalidomide or not)

^b^Index date was defined as the date at which the patient entered the cohort (= at 2L treatment initiation)

^c^Including all patients with at least one comorbidity; note that patient numbers across comorbidities do not sum to the total number of patients because one patient could have multiple comorbidities

*Abbreviations: ASCT,* autologous stem cell transplant; *IQR,* interquartile range; *MM,* multiple myeloma; *SD,* standard deviation; *2L,* second line

### Treatment patterns by ASCT status at 1L

**Fig. 2:**
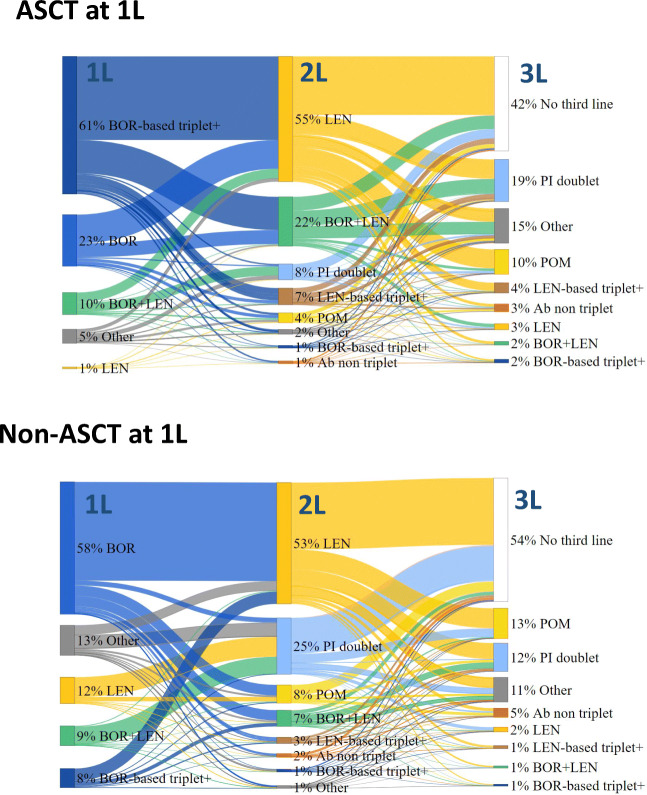
**Treatment patterns across lines of treatment for patients with RRMM by ASCT status at 1L.** PI doublet: bortezomib-, carfilzomib-, ixazomib-based doublet – BOR-based triplet+: BOR+THA, BOR+DAR – LEN-based triplet+: LEN+CAR, LEN+IXA, LEN+DAR – Ab non-triplet: DAR without any other drugs of interest – Others: specific chemotherapy (at least 1 drug of interest) not part of study regimens of interest or unspecified chemotherapy. Note: BOR-based doublet is included in PI doublet; however, given that PI doublet at 1L only contains BOR-doublet, BOR-doublet alone is represented at 1L. At 2L and 3L, BOR-based doublet is the predominant PI doublet, representing 75% to 97% of PI doublet regimens. *Abbreviations: Ab*, antibody; *ASCT*, autologous stem cell transplant; *BOR*, bortezomib; *CAR*, carfilzomib; *DAR*, daratumumab; *IXA*, ixazomib; *LEN*, lenalidomide; *PI*, proteasome inhibitor; *RRMM*, relapsed or refractory multiple myeloma; *THA*, thalidomide; *1L*, first line; *2L*, second line; *3L*, third line

In both the ASCT and non-ASCT subgroups, the majority of patients received bortezomib-based regimens at 1L (84 and 64% of patients, respectively). These were bortezomib-based doublet or triplet regimens that excluded lenalidomide: triplet regimens were most common in the ASCT subgroup (61% of patients), whereas doublet regimens were more common in the non-ASCT subgroup (58% of patients; Fig. 2). The most frequently used 2L treatment was lenalidomide-based doublet regimens in both ASCT and non-ASCT subgroups (55 and 53% of patients, respectively). The second most common 2L regimen was a combination of bortezomib and lenalidomide for the ASCT subgroup (22% of patients) and a PI-based doublet (in particular, bortezomib-based doublet) for the non-ASCT subgroup (25% of patients). At third-line (3L) treatment, PI-based doublets (predominately bortezomib-based doublets) were the most common regimens used in the ASCT subgroup (19% of patients), followed by pomalidomide (11% of patients). In the non-ASCT subgroup, pomalidomide was the most common 3L treatment (13% of patients), followed by PI-based doublets (predominately bortezomib-based doublets) (12% of patients; Fig. 2).

**Fig. 3:**
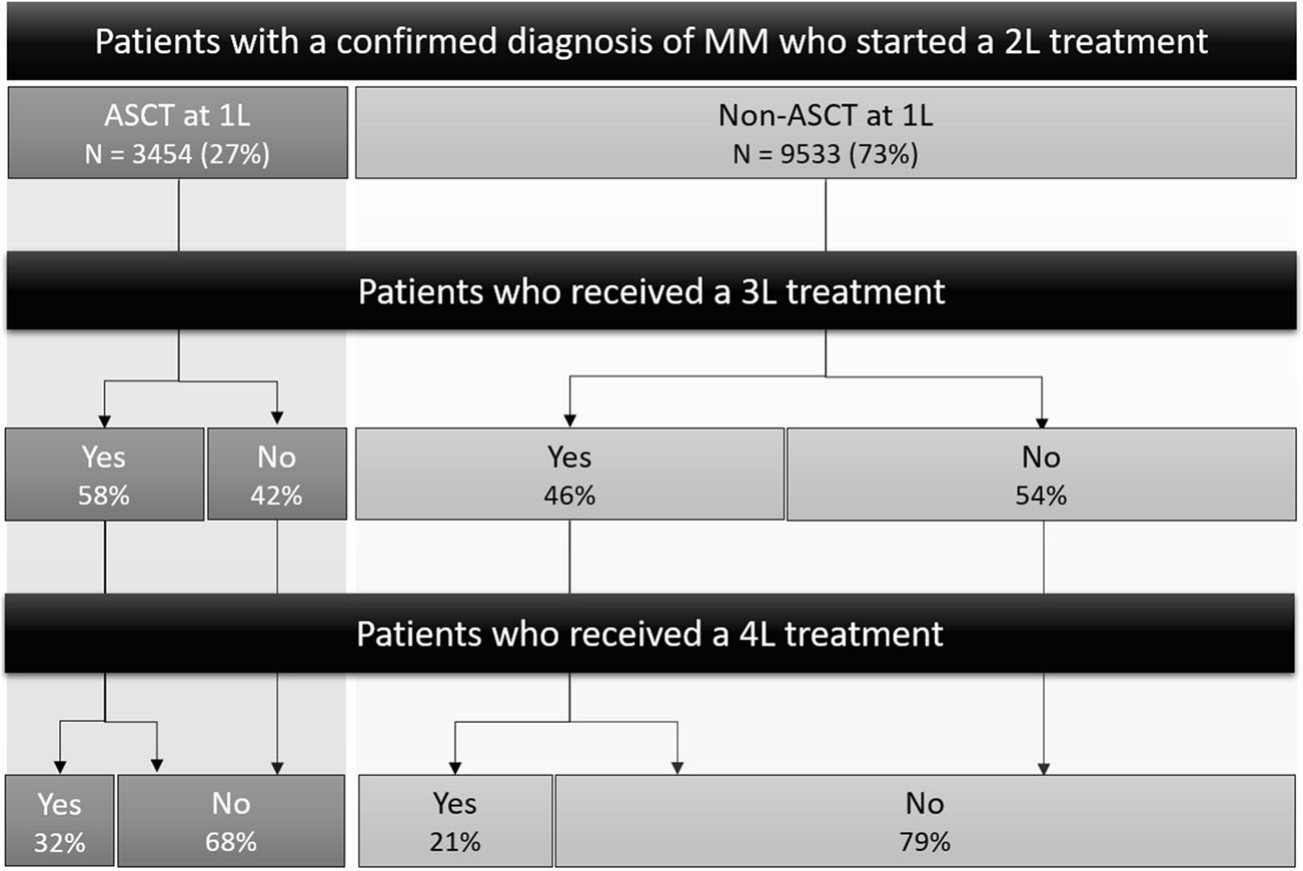
**Number of lines of treatment for patients with RRMM by ASCT status at 1L***. Abbreviations: ASCT*, autologous stem cell transplant; *RRMM*, relapsed or refractory multiple myeloma; *1L*, first line; *2L*, second line; *3L*, third line; *4L*, fourth line

Overall, a higher proportion of patients received a 3L and fourth-line (4L) treatment in the ASCT subgroup than in the non-ASCT subgroup during follow-up (3L: 58% vs 46%; 4L: 32% vs 21%; Fig. 3).

### Treatment patterns by lenalidomide status at 2L

**Fig. 4:**
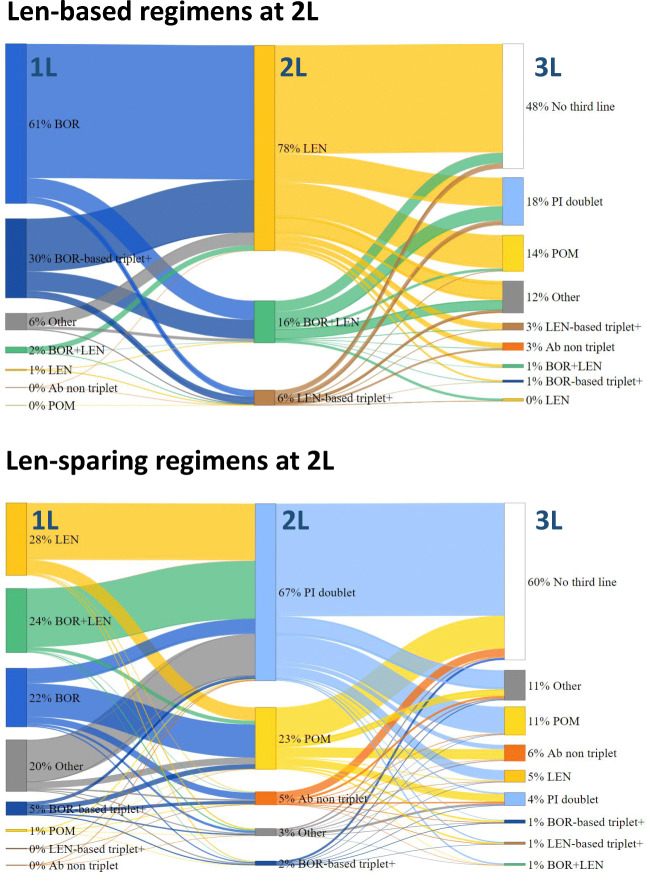
**Treatment patterns across lines of therapy for patients with RRMM by lenalidomide-based regimens status at 2L.** PI doublet: bortezomib-, carfilzomib, ixazomib-based doublet – BOR-based triplet+: BOR+THA, BOR+DAR – LEN-based triplet+: LEN+CAR, LEN+IXA, LEN+DAR – Ab non-triplet: DAR without any other drugs of interest – Others: specific chemotherapy (at least 1 drug of interest) not part of study regimens of interest or unspecified chemotherapy. Note: BOR-based doublet is included in PI doublet; however, given that PI doublet at 1L only contains BOR-doublet, BOR-doublet alone is represented at 1L. At 2L and 3L, BOR-based doublet is the predominant PI doublet, representing 75 to 97% of PI doublet regimens. *Abbreviations: Ab*, antibody; *BOR*, bortezomib; *CAR*, carfilzomib; *DAR*, daratumumab; *IXA*, ixazomib; *LEN*, lenalidomide; *PI*, proteasome inhibitor; *RRMM*, relapsed or refractory multiple myeloma; *THA*, thalidomide; *1L*, first line; *2L*, second line; *3L*, third line

At 1L, most patients (93%) in the lenalidomide subgroup received a bortezomib-based regimen; 61% of patients in this subgroup received a bortezomib-based doublet at 1L (Fig. 4). At 2L, most patients in the lenalidomide subgroup received a lenalidomide-based doublet regimen (78% of patients). In the lenalidomide-sparing subgroup, most patients received either a bortezomib-based or a lenalidomide-based regimen at 1L (Fig. 4). The most common 2L treatment in the lenalidomide-sparing subgroup was a PI-based doublet regimen (predominately bortezomib-based doublets), which was given to 67% of patients.

### Mortality

In our study population of patients with RRMM who were initiated on a 2L treatment, 40% died during the follow-up period (mean [SD] follow-up time 18.5 [15.3] months, median follow-up time 14.6 months). By subgroup, the proportions of patients who died during follow up were 28% in the ASCT subgroup, 45% in the non-ASCT subgroup, 39% in the lenalidomide subgroup, and 44% in the lenalidomide-sparing subgroup.

**Fig. 5:**
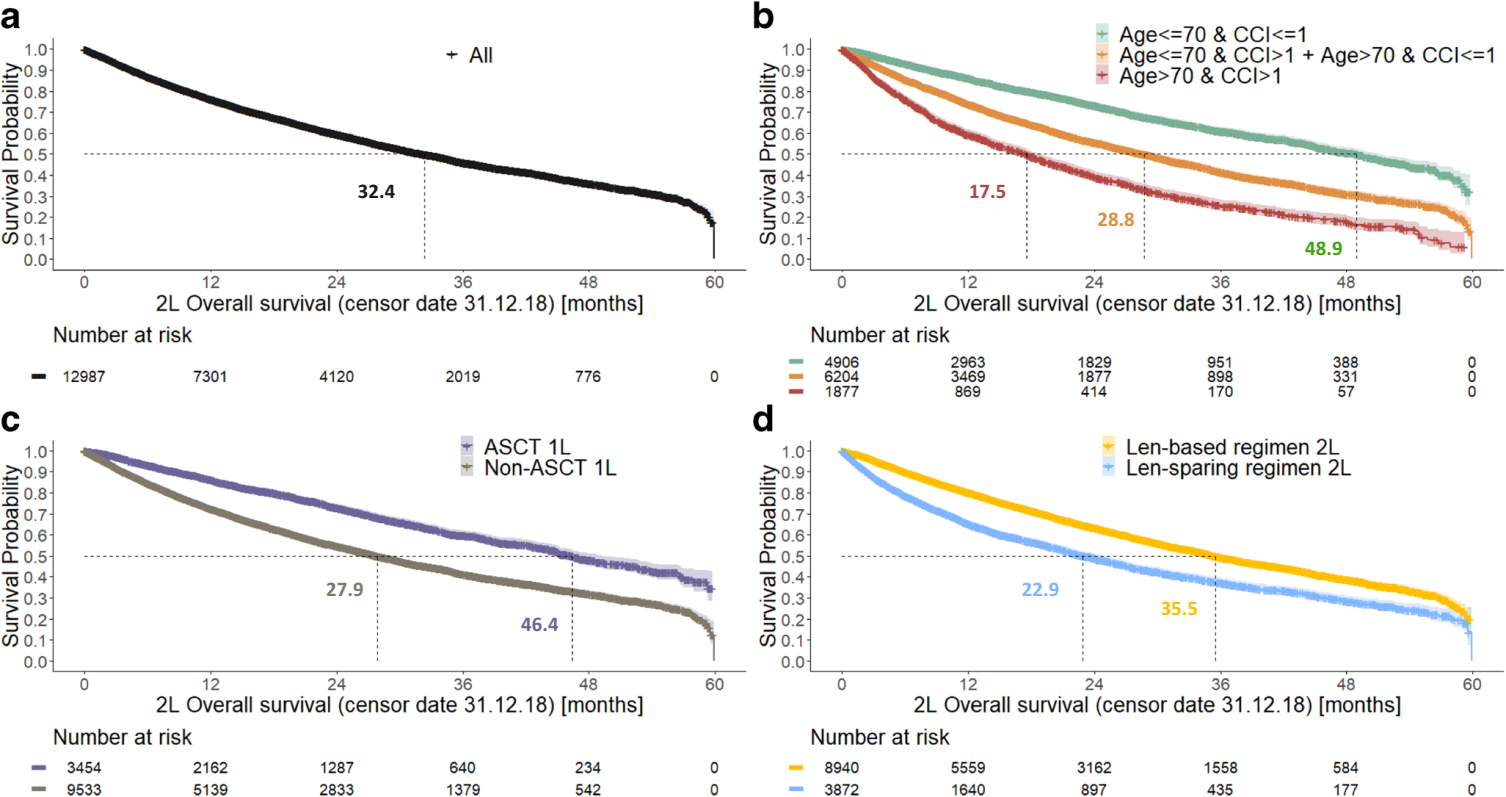
**Overall survival from initiation of 2L treatment: overall cohort (a), by age and CCI group (b), by ASCT status at 1L (c), and by lenalidomide status at 2L (d).** *Abbreviations: ASCT*, autologous stem cell transplant; *CCI*, Charlson comorbidity index; *1L*, first line; *2L*, second line

Overall, mortality rate was 26.1/100 person-years and was almost twice as high for the non-ASCT subgroup as for the ASCT subgroup (30.1/100 person-years vs 16.5/100 person-years) (Table 3, Fig. 5). Patients in the ASCT subgroup were younger and had fewer comorbidities than those in the non-ASCT subgroup. When considering mortality rate by age and/or CCI, it remained higher for the non-ASCT subgroup and in particular among patients with a high CCI (> 1) (Table 3).

**Table 3:** Mortality rate during follow-up (per 100 person-years), from 2L onwards, overall, and according to ASCT status at 1L or lenalidomide-based regimens status at 2L

	Mortality rate during follow-up (per 100 person-years)	
Overall		
Overall	26·1 (*n* = 12987)	
By age and CCI		
Age ≤ 70 and CCI ≤ 1	16·2 (*n* = 4906)	
Age ≤ 70 and CCI > 1	30·1 (*n* = 1814)	
Age > 70 and CCI ≤ 1	29·3 (*n* = 4390)	
Age > 70 and CCI > 1	47·7 (*n* = 1877)	
By ASCT status at 1L, overall and according to age and CCI		
	ASCT	Non-ASCT
Subgroup according to ASCT status at 1L	16·5 (*n* = 3454)	30·1 (*n* = 9533)
Age group (at index date)		
Age ≤ 70	16·5 (*n* = 3309)	22·8 (*n* = 3411)
Age > 70	18·2 (*n* = 145)	34·4 (*n* = 6122)
Charlson comorbidity index (CCI)		
CCI ≤ 1	14·8 (*n* = 2807)	25·7 (*n* = 6489)
CCI > 1	25·3 (*n* = 647)	41·9 (*n* = 3044)
Age group + CCI		
Age ≤ 70 and CCI ≤ 1	14·8 (*n* = 2687)	18·2 (*n* = 2219)
Age ≤ 70 and CCI > 1	25·0 (*n* = 622)	33·2 (*n* = 1192)
Age > 70 and CCI ≤ 1	15·1 (*n* = 120)	29·7 (*n* = 4270)
Age > 70 and CCI > 1	36·7 (*n* = 25)	47·8 (*n* = 1852)
By lenalidomide status at 2L		
	Lenalidomide-based regimen	Lenalidomide-sparing regimen
Subgroup according to lenalidomide status at 2L	23·0 (*n* = 8940)	36·4 (*n* = 3872)

*Abbreviations: ASCT,* autologous stem cell transplant; *CCI,* Charlson comorbidity index; *1L,* first line; *2L* second line

Patients treated with lenalidomide-based regimens at 2L had lower mortality rate than those treated with lenalidomide-sparing regimens (23.0/100 person-years vs 36.4/100 person-years; Table 3, Fig. 5). Further analysis using a multivariate-adjusted Cox regression model showed that patients treated with lenalidomide-sparing regimens at 2L had an approximately 60% higher risk of death than those who received lenalidomide-based regimens at 2L (hazard ratio [95% CI] 1.6 [1.5–1.8]) (Supplementary material 4).

Overall, median (95% CI) survival time from 2L initiation was 32.4 (31.2–33.6) months; survival time decreased from 48.9 (46.4–51.1) to 28.5 (25.9–31.1), 28.9 (27.4–30.3), and 17.5 (16.0–18.9) months for the subgroups “age ≤ 70 and CCI ≤ 1,” “age ≤ 70 and CCI > 1,” “age > 70 and CCI ≤ 1,” and “age > 70 and CCI > 1,” respectively. Survival also decreased from 46.4 (44.5–49.2) months for patients in the ASCT subgroup to 27.9 (27.0–29.1) months for those in the non-ASCT subgroup and from 35.5 (34.5–37.1) months in patients receiving a lenalidomide-based regimen at 2L to 22.9 (21.7–24.8) months in those receiving a lenalidomide-sparing regimen at 2L (Fig. 5).

## Discussion

This study describes a diverse treatment landscape for RRMM in a nationwide cohort of 12987 patients treated with approved novel therapies between 2014 and 2018 in France. A few treatments, however, emerged as the preferred choices at each treatment line. At 1L, most patients were treated with bortezomib, whereas lenalidomide was the most commonly used agent at 2L. PI-based doublet regimens, predominately bortezomib-based doublets, and pomalidomide were the most common 3L treatments. In this cohort study, 40% of the patients died during the follow-up period; mortality rate was 26.1/100 person-years, with a median survival time from 2L initiation of 32.4 months. Compared with the overall cohort, survival was shorter for patients who did not receive an ASCT at 1L, those receiving a lenalidomide-sparing regimen at 2L, older patients (≥ 70 years) and those with multiple comorbidities.

Although there continues to be no standard of care for RRMM in Europe [32], recent studies show that lenalidomide is often the most common treatment, and PI-based regimens, including triplets, are increasingly being prescribed [21, 33]. Practice patterns in France differ from the rest of Europe, and only a few studies have reported on recent treatment patterns in RRMM that are specific to the French population [19, 20]. These studies align with the findings of the current study in terms of the most common 2L treatments; however, treatment patterns appear to diverge at 3L treatment. [19]Treatment choice in the RRMM landscape can be influenced by many patient- and disease-related factors [1]. In our study, treatment regimens were varied, particularly at 3L and beyond, potentially reflecting the wide variability in patient characteristics and prior response. Transplantation at 1L also appears to influence subsequent treatment choices: triplet regimens were more frequently prescribed to patients who received an ASCT at 1L than to those who did not. Studies have shown that younger patients, with fewer comorbidities, are likely to tolerate more lines of treatment (including ASCT) and more aggressive regimens [1, 22, 34, 35]. Access to and reimbursement of treatments also influences treatment patterns [1]. In our study, some recent therapies were not available in France except in “Temporary Authorization for Use” (ATU). Carfilzomib became fully available in July 2018 (previously in ATU and compassionate use thereafter since 2016) and ixazomib in October 2018. During the study, daratumumab and elotuzumab were not reimbursed, while pomalidomide was. Despite several agents not being reimbursed, regimens including these more recently available drugs have been captured in our study, particularly at 3L and beyond. Our findings suggest a potential gap in treatment for patients in France, given that recent clinical trials have shown that agents such as carfilzomib, daratumumab, elotuzumab, and ixazomib can result in substantially improved progression-free survival in RRMM [7–12, 15, 16]. Furthermore, there is evidence that use of these drugs in earlier treatment lines can produce a greater depth of response and further improve outcomes [36].

Our study evaluated treatments for RRMM in a real-world setting, where patient populations are more heterogeneous than clinical trial populations; routine clinical care may differ from the more rigorous protocols followed in a clinical trial setting [37]; and patient outcomes have been observed to differ from those achieved in clinical trial settings for MM [25, 38]. Patients in our cohort study were similar in age to clinical trial populations [7, 9–12, 15, 16], and 40% of patients died during the follow-up period, similar to other real-world French studies [20]. Patients in our study who received a lenalidomide-sparing regimen at 2L had a lower median survival time than those receiving a lenalidomide-based regimen; adjusted analyses confirmed this relationship between use of lenalidomide-sparing regimens at 2L and increased risk of death. Among patients treated with lenalidomide-sparing regimens at 2L, half received lenalidomide-based regimens at 1L and may have had lenalidomide-refractory RRMM and a poor prognosis. These findings highlight the need for new effective therapeutic strategies for patients who cannot receive lenalidomide-based regimens at 2L [39].

Limitations of this study include those inherent to the use of retrospective administrative claims data and limitations associated with missing data. However, we had access to multiple linked data sources (primary care, hospital, pharmacy data, and central death registrations), which greatly improves exposure and outcome ascertainment. Furthermore, the SNDS includes data on 99% of the French population, enabling the analysis of a large and representative cohort of patients with RRMM. For patients receiving a newly approved treatment available from 2018 onwards, sample sizes were low and exposure to such treatments and follow-up were limited [39]. The current study also did not capture clinical trial participation and information on the line of therapy for such patients may not be precise.

This real-world analysis illustrates the dynamic MM treatment paradigm and provides useful information for payers making decisions about reimbursement options and providers evaluating choice of treatment regimens in order to optimize the management of RRMM. Survival data from this study suggest that there remains a need to improve real-world outcomes, particularly among selected patient populations.

## Supplementary Information

ESM 1 (PDF 839 kb).

## Data Availability

Data were shared by the French national health insurance: “Caisse Nationale d’Assurance Maladie” (CNAM).

## Code availability

All statistical analyses were conducted using SAS® Enterprise Guide version 7.15. Graphical representation was carried out using R version 3.6.2. Specific code may be available upon request.
