# Supplementary material for: Survival and treatment patterns of patients with relapsed or refractory multiple myeloma in France — a cohort study using the French National Healthcare database (SNDS)
Source: Ann Hematol. 2021 Apr 21;100(7):1825–36. doi: 10.1007/s00277-021-04522-y (PMC8195931; doi:10.1007/s00277-021-04522-y)
Supplement: Supplementary file 1 — (PDF 839 kb). [file 277_2021_4522_MOESM1_ESM.pdf]

Annals of Hematology

Survival and treatment patterns of patients with relapsed or refractory multiple myeloma in France – a cohort study using the French National Healthcare database (SNDS)

Cyrille Touzeau[1,2,3,*] • Nadia Quignot[4] • Jie Meng[5] • Heng Jiang[4] • Artak Khachatryan[6] • Moushmi Singh[7] • Vanessa Taieb[7] • Jean-Vannak Chauny[8] • Gaëlle Désaméricq[8]

[1]Service d'hématologie clinique, Hôtel Dieu, Nantes, France; [2]CRCINA, INSERM, CNRS, Université d'Angers, Université de Nantes, France; [3]Site de Recherche Intégrée sur le Cancer (SIRIC) « ILIAD », Nantes, France; [4]Certara Evidence & Access, Paris, France; [5]Certara Evidence & Access, Lorrach, Germany; [6]Certara Evidence & Access, London, United Kingdom; [7]Amgen Ltd, London, United Kingdom, [8]Amgen SAS, Boulogne-Billancourt, France

*Corresponding author, Cyrille.TOUZEAU@chu-nantes.fr

Supplementary material 1. Study design

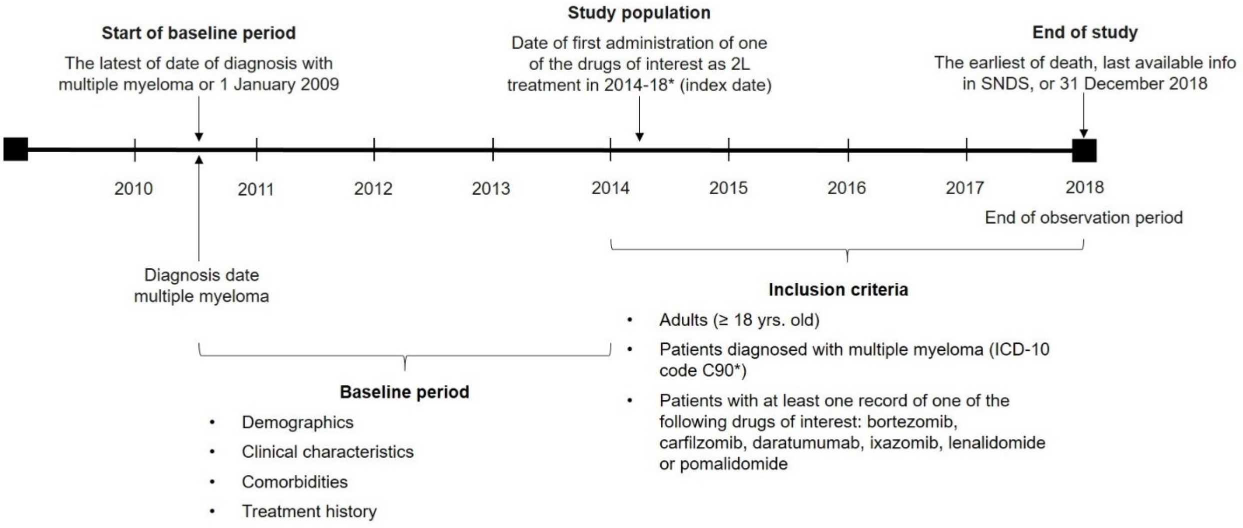

Treatments of interest were selected since they are predominantly the new drugs that received EMA marketing authorization within the last five years (2014-2018) (Cavo et al, 2018). As elotuzumab is not prescribed and reimbursed in France, it was not tracked**.**

Cavo M, Terpos E, Bargay J, Einsele H, Cavet J, Greil R, et al. The multiple myeloma treatment landscape: international guideline recommendations and clinical practice in Europe. Expert Review of Hematology. 2018 2018/03/04;11(3):219-37.

Supplementary material 2. List of codes

<u>ICD-10 diagnosis code to identify multiple myeloma patients</u>

Type	Label	Code
ICD-10	Multiple myeloma	C90*

* This code was used to identify MM patients with at least one occurrence of the diagnosis (either principal, associated or related) for a hospitalization occurring during the study observation period (Palmaro et al. 2017). This code was also searched in the long-term illness database to have the date of first registration and thus to know whether the patient had been diagnosed during the study period.

Palmaro A, Gauthier M, Conte C, Grosclaude P, Despas F, Lapeyre-Mestre M. Identifying multiple myeloma patients using data from the French health insurance databases: Validation using a cancer registry. Medicine (Baltimore). 2017;96(12):e6189.

<u>List of ATC codes for drugs of interest</u>

Agent	ATC Code	UCD codes
Bortezomib	L01XX32	3400893189108, 3400892600109
Carfilzomib	L01XX45	3400894207320, 3400894207498, 3400894138389
Daratumumab	L01XC24	3400894178712, 3400894178880
Ixazomib	L01XX50	3400894262787, 3400894262848, 3400894262909
Lenalidomide	L04AX04	3400892981130, 3400892981369, 3400894056782, 3400894082927, 3400892981420, 3400892981598, 3400894083009
Pomalidomide	L04AX06	3400893957967, 3400893958049, 3400893958100, 3400893958278

<u>List of ATC codes for other drug</u>

Agent	ATC Code
Thalidomide	L04AX02

List of ICD-10 diagnosis codes, DRG and CCAM procedure codes to identify chemotherapy

Type	Label	Code
ICD-10	Chemotherapy session for neoplasm	Z51.1
DRG	Chemotherapy, series	28Z07Z
	Chemotherapy for other neoplasm, short term	17M06T
	Chemotherapy for other neoplasm, level 1	17M061
	Chemotherapy for other neoplasm, level 2	17M062
	Chemotherapy for other neoplasm, level 3	17M063
	Chemotherapy for other neoplasm, level 4	17M064
CCAM	Locoregional intra-arterial injection of neoplastic drug by implanted device	ZZLF900

List of DRG and CCAM procedure codes to identify autologous stem cell transplant (ASCT)

Type	Label	Code
DRG	Autografts of hematopoietic stem cells	27Z03Z
	Grafts of hematopoietic stem cells, for ambulatory patients	27Z04J
CCAM	Intravenous injection of cell therapy product for autograft	FELF010

Supplementary material 3. Regimen definitions

**Regimen Group***	**Mode of action group**
Carfilzomib doublet<br>Bortezomib doublet<br>Ixazomib doublet	Proteosome inhibitor-based non-triplet regimens
Bortezomib/Thalidomide<br>Bortezomib/Daratumumab triplet+	Proteosome inhibitor-based triplet regimens
Lenalidomide doublet<br>Pomalidomide doublet	IMiD-based non-triplet regimens
Lenalidomide/Carfilzomib triplet+<br>Lenalidomide/Bortezomib triplet+<br>Lenalidomide/Daratumumab triplet+<br>Lenalidomide/Ixazomib triplet+<br>Pomalidomide/Bortezomib triplet+<br>Pomalidomide/Daratumumab triplet+	IMiD-based triplet regimens
Daratumumab mono/doublet	Antibody non-triplet regimens

*Patients may have received another anti-cancer drug besides the seven molecules of interest. Other anti-cancer drugs include: cyclophosphamide, doxorubicin, thalidomide, bendamustine, cisplatin, etoposide, liposome-encapsulated doxorubicin, melphalan, pegylated liposomal doxorubicin.

Non-triplet regimens were determined by use of one drug of interest without any other drugs of interest. Triplet+ regimens included regimens in which two drugs of interest were used concomitantly; drugs of a same combination should be initiated within 90 days.

Line of therapy was defined according to an algorithm using the earliest diagnosis date of MM and treatment records. After identification of the earliest MM diagnosis date, all treatment records following this date were selected. It was assumed that start date of treatment was the recorded date of dispensation. The treatment period was defined as the timeframe between first and last dispensation date without a gap of 105 days between these 2 events; if the same treatment was restarted within any timeframe, it was considered as the same line of treatment. The combination of ASCT together with induction and maintenance chemotherapy was considered as a unique line of therapy. The line number was then defined. The date for the regimen start date after 01/01/2014 was then identified.

Supplementary material 4. Cox regression for all-cause mortality among the RRMM patients at index date (= initiation of 2L treatment)

Univariate Cox regression models for all-cause mortality among the RRMM patients at index date (= initiation of 2L treatment)

Variables	Frequency (*n* = 12812)	Univariate models HR (95% CI)	P value
**Treatment at 2L initiation (*n*, %)**			
Lenalidomide-based regimen	8940 (70%)	Reference	
Lenalidomide-sparing regimen	3872 (30%)	1.6 (1.5, 1.7)	p<0.0001
**Age at initiation of 2L treatment (years) (*n*, %)**			
≤ 60	2450 (19%)	Reference	
61 – 65	1667 (13%)	1.1 (1.0, 1.2)	p=0.0765
66 – 70	2454 (19%)	1.3 (1.2, 1.4)	p<0.0001
71 – 75	2185 (17%)	1.5 (1.4, 1.7)	p<0.0001
76 – 80	2056 (16%)	1.9 (1.7, 2.1)	p<0.0001
> 80	2000 (16%)	2.7 (2.4, 2.9)	p<0.0001
**Time from MM diagnosis to initiation of 2L treatment (*n*, %)**			
[0,8] months	2933 (23%)	Reference	
(8, 18] months[#]	2405 (19%)	1.3 (1.2, 1.4)	p<0.0001
(18, 31] months	2358 (18%)	1.2 (1.1, 1.3)	p=0.0011
(31, 57] months	2567 (20%)	0.9 (0.8, 0.9)	p=0.0006
> 57 months	2549 (20%)	0.8 (0.7, 0.9)	p<0.0001
**Sex (*n*, %)**			
Female	5893 (46%)	Reference	
Male	6919 (54%)	1.0 (1.0, 1.1)	p=0.7726
**Autologous stem cell transplantation prior to index date (*n*, %)**			
No	9413 (73%)	Reference	
Yes	3399 (27%)	0.5 (0.5, 0.6)	p<0.0001
**Comorbidities (*n*, %)**			
Hypertension (reference: without the disease)	5581 (44%)	1.4 (1.3, 1.5)	p<0.0001
Dementia (reference: without the disease)	979 (8%)	1.4 (1.3, 1.6)	p<0.0001
Diabetes (reference: without the disease)	1690 (13%)	1.3 (1.2, 1.4)	p<0.0001
Moderate to severe renal disease (reference: without the disease)	1276 (10%)	1.8 (1.6, 1.9)	p<0.0001
Any tumor[&] (reference: without the disease)	1946 (15%)	1.5 (1.4, 1.7)	p<0.0001

[#] (8,18] means domain have range is 8 to 18, including 18 but not 8

[&] including lymphoma, leukemia except for malignant neoplasm of skin, and metastatic solid tumor

Abbreviations: HR, Hazard Ratio*; MM*, multiple myeloma; *RRMM*, relapsed or refractory multiple myeloma; *2L*, second line

Adjusted Cox regression model with stratification[#] for all-cause mortality among the RRMM patients at index date (= initiation of 2L treatment)

**Variables**	**Frequency (*n* = 12812)**	**Adjusted models[*] HR (95% CI)**	**P value**
**Treatment at 2L initiation (*n*, %)**			
Lenalidomide-based regimen	8940 (70%)	Reference	
Lenalidomide-sparing regimen	3872 (30%)	1.6 (1.5, 1.8)	$p<0.0001$

[#] The adjusted model is stratified by ASCT prior to index date, time from MM diagnosis to initiation of 2L treatment, and moderate to severe renal disease.

[*] Covariates in the model include: treatment at 2L initiation, age at initiation of 2L treatment, sex, hypertension, dementia, diabetes, any tumor (including lymphoma, leukemia except for malignant neoplasm of skin, and metastatic solid tumor)

Abbreviations: HR, Hazard Ratio*; MM*, multiple myeloma; *RRMM*, relapsed or refractory multiple myeloma; *2L*, second line
